# Relapsing Polychondritis following PD-1 Blockade by an Immune Checkpoint Inhibitor

**DOI:** 10.31662/jmaj.2023-0071

**Published:** 2023-09-20

**Authors:** Tomoyuki Mutoh, Sonoko Chikamatsu, Takatsuna Sasaki, Hiroto Seino, Kazuhiro Sakamoto, Masataka Kudo

**Affiliations:** 1 Department of Rheumatology, Osaki Citizen Hospital, Osaki, Japan; 2 Department of Medical Oncology, Osaki Citizen Hospital, Osaki, Japan; 3 Department of Otolaryngology, Osaki Citizen Hospital, Osaki, Japan; 4 Department of Plastic and Reconstructive Surgery, Osaki Citizen Hospital, Osaki, Japan; 5 Department of Pathology, Osaki Citizen Hospital, Osaki, Japan; 6 Department of Nephrology and Endocrinology, Osaki Citizen Hospital, Osaki, Japan

**Keywords:** relapsing polychondritis, immune checkpoint inhibitors, immune-related adverse events, programmed cell death protein-1 inhibitor, pembrolizumab

## Abstract

Immune-related adverse events (irAEs) mimicking rheumatic diseases are observed in 1.5%-22% of patients receiving cancer therapy with immune checkpoint inhibitors (ICIs). Relapsing polychondritis (RP) is a rare autoimmune disease mainly involving auricle, nose, and airway cartilage inflammation. However, knowledge regarding RP as an irAE is scarce. Pembrolizumab, a type of ICI that regulates the programmed cell death protein-1 (PD-1), is used in patients whose cancer cannot be cured with surgery or radiation therapy. We report the first case of pembrolizumab-induced RP with isolated auricular lesions resolved without immunosuppressants. A 49-year-old man with lower lip cancer underwent surgical resection followed by reconstruction. Histopathological investigation confirmed the diagnosis of squamous cell carcinoma. Since multiple metastases 6 months post-surgery rendered the carcinoma inoperative, pembrolizumab was initiated, improving lymph node involvement. However, 4 months later, the patient developed rapidly progressive swelling and pain in both auricles. While no pathogen was detected, C-reactive protein levels were elevated (11.21 mg/dL). Computed tomography (CT) showed swelling of the bilateral auricles; the biopsy of the right auricle revealed cartilage destruction by infiltration of surrounding granulation tissue. Since these characteristic findings were not observed before pembrolizumab was initiated, we clinically diagnosed the patient with RP induced by pembrolizumab.

The swelling of the auricles resolved spontaneously 1 month after pembrolizumab discontinuation. ^18^F-fluorodeoxyglucose (^18^F-FDG)-positron emission tomography/CT revealed no ^18^F-FDG uptake in reduced auricular lesions. On re-administration of pembrolizumab to maintain antitumor immunity, both auricles swelled again, and pembrolizumab was switched to paclitaxel, considering the risk of tracheobronchial chondritis. Although no recurrence of auricular chondritis was observed, the patient died from cancer progression 8 months after paclitaxel administration. RP can occur as a rheumatic irAE in patients receiving anti-PD-1 therapy, and a literature review with retrospective analysis indicates that PD-1 inhibition-induced RP is unusual and atypical.

## Introduction

Using immune checkpoint inhibitors (ICIs) in cancer therapy has increased the incidence of immune-related adverse events (irAEs) mimicking rheumatic diseases ^(1)^. Relapsing polychondritis (RP) is a rare rheumatic disease mainly affecting the auricle, nose, and tracheobronchial cartilage ^(2)^. To date, knowledge regarding RP as an irAE is scarce. Herein, we report the first case of pembrolizumab-induced RP with isolated auricular lesions, a programmed cell death protein-1 (PD-1) inhibitor. We also provide further evidence on the disease phenotype and pathophysiological mechanisms through literature review and clarify the incidence of RP associated with PD-1 inhibition at our institution in a retrospective analysis.

## Case Report

**Figure 1.**
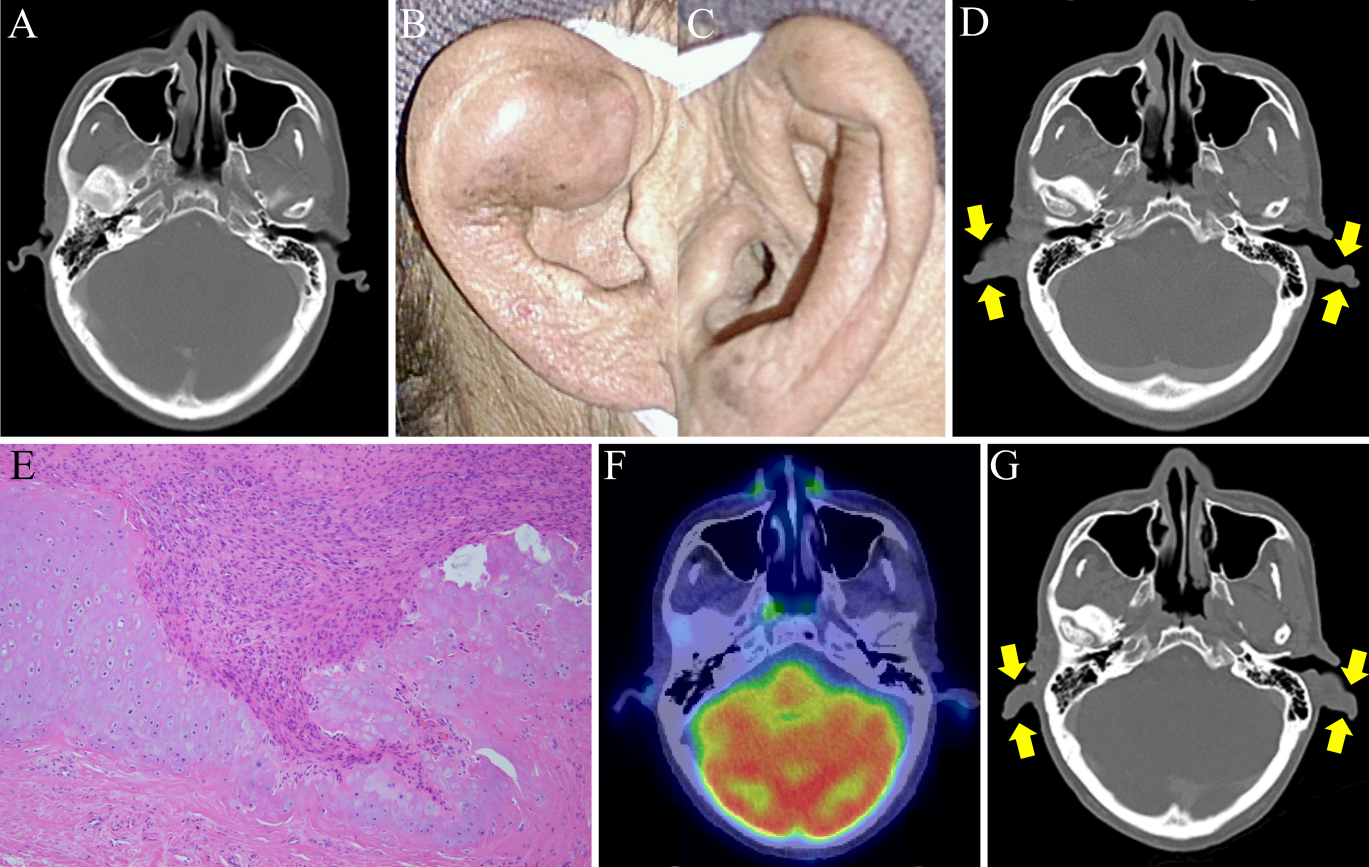
Auricular involvement in a patient with relapsing polychondritis induced by pembrolizumab, a programmed cell death protein-1 inhibitor. (A) No enlargement in the bilateral auricles was observed in CT before pembrolizumab therapy. (B-D) Swelling of the bilateral auricles developed 4 months after pembrolizumab therapy (B, right ear; C, left ear; D, yellow arrows in CT indicate auricular involvement). (E) Hematoxylin-eosin-stained images of the right auricle reveal cartilage destruction due to infiltration of the surrounding granulation tissue (magnification, 100×). (F) Lack of ^18^F-FDG uptake in the regressed swelling of bilateral auricles on ^18^F-FDG-PET/CT 1 month after interruption of pembrolizumab therapy. (G) CT showing relapsing auricular involvement after three cycles of pembrolizumab therapy. Yellow arrows indicate the reswelling of the bilateral auricles. ^18^F-FDG-PET/CT, ^18^F-fluorodeoxyglucose-positron emission tomography/computed tomography.

A 49-year-old man with an unremarkable medical history presented with progressive swelling of the lower lip over 1.5 months. Physical examination revealed a multilocular mass with elastic hardness throughout the lower lip, extending to the oral cavity, and painless cervical lymphadenopathy. ^18^F-fluorodeoxyglucose-positron emission tomography/computed tomography (^18^F-FDG-PET/CT) revealed ^18^F-FDG uptake in the lower lip mass and bilateral submandibular nodes. The patient underwent surgical resection with lymphadenectomy, then reconstruction with a thigh flap. Histopathological investigation of the surgical specimen confirmed the diagnosis of squamous cell carcinoma. Metastases to the right pleura, multiple pulmonary lesions, and left hilar lymph nodes developed 6 months post-surgery. Since multiple metastases rendered the carcinoma inoperative, pembrolizumab was initiated, improving lymph node involvement. Pre-pembrolizumab administration CT showed no abnormalities in the auricles (Figure 1A). However, 4 months later, the patient developed rapidly progressive swelling and pain in both auricles (Figure 1B and 1C). While C-reactive protein values were elevated (11.21 mg/dL), screening for infection, immunological tests for rheumatoid factor, anti-nuclear antibody, and anti-neutrophil cytoplasmic antibodies were negative. With the swelling of the bilateral auricles (Figure 1D) without tracheobronchial thickening as confirmed by CT, the biopsy of the right auricle revealed cartilage destruction by infiltration of surrounding granulation tissue (Figure 1E). Although there are reports of RP associated with malignancy, this patient’s symptoms are considered immune-related adverse events based on the clinical course. Thus, the patient was diagnosed with pembrolizumab-induced RP. The swelling of the auricles resolved spontaneously 1 month after discontinuing pembrolizumab. PET/CT also revealed no ^18^F-FDG uptake in reduced auricular lesions (Figure 1F). Pembrolizumab was readministered to maintain antitumor immunity. However, after three cycles of pembrolizumab, both auricles swelled again (Figure 1G). Pembrolizumab was switched to paclitaxel, considering the risk of tracheobronchial chondritis, a potentially life-threatening condition. Although no recurrence of auricular chondritis was observed, the patient died from cancer progression 8 months after paclitaxel administration.

## Discussion

To our knowledge, this is the first case of pembrolizumab-induced RP with isolated auricular lesions. A PubMed search for English articles describing the clinical characteristics of ICI-induced chondritis published until March 2023 yielded only five cases treated with nivolumab, another anti-PD-1 antibody (Table 1) ^(3), (4), (5), (6), (7)^. As shown in Table 1, all patients belonging to 49-72 years age group had 50% of having hypopharyngeal carcinoma. The median duration between chondritis onset and ICI initiation was 4.5 months, and ICIs were restricted to PD-1 inhibition. All reported cases, excluding the present case wherein the affected cartilage was auricular, mainly included isolated tracheobronchial chondritis. Discontinuing ICIs with varied immunosuppressants improved the symptoms ^(3), (5), (6)^. Notably, all previous cases were from Japan, suggesting that genetic factors could be associated with chondritis development and human leukocyte antigen (HLA). HLA-DR4 has been identified as a major risk allele, and the pathological process includes autoimmunity by producing multiple inflammatory cytokines, degradative enzymes, and autoantibodies targeting cartilage components ^(2)^. Although the precise pathophysiology underlying irAEs remains unclear, inhibiting negative signals on activated immune cells could contribute to increased autoreactive T-cell activity and elevated levels of preexisting autoantibodies and inflammatory cytokines ^(8)^. These aberrant immune reactions triggered by ICIs might develop RP in genetically predisposed individuals. Further research is warranted to determine clinical features and disease mechanisms.

**Table 1.** Summary of Demographics and Clinical Characteristics of 6 Patients with ICI-Induced Chondritis.

Case	Age/Sex	Pre-existing malignancy	ICI	Duration from ICI initiation to chondritis onset	Chondritis involvement			Therapies for chondritis	ICI cessation	Outcome of chondritis	Country, (ref)
					Auricle	Nose	Airway				
1	72/M	Hypopharyngeal carcinoma	Nivolumab	4 months	−	−	+	PSL (0.5 mg/kg/day) after HC (300 mg)	+	Improvement after relapse	Japan, ^(3)^
2	68/M	Esophageal carcinoma	Nivolumab	5 months	−	−	+	PSL (50 mg/day)	NA	Improvement	Japan, ^(4)^
3	72/M	Mandibular cancer	Nivolumab	3 months	−	+	+	Inhaled GC	+	Improvement	Japan, ^(5)^
4	71/M	Hypopharyngeal carcinoma	Nivolumab	10^th^ course of ICI	−	−	+	PSL (60 mg/day)	+	Improvement	Japan, ^(6)^
5	66/M	Hypopharyngeal cancer	Nivolumab	24^th^ course of ICI	−	−	+	High-dose GC, MTX, TCZ	NA	Improvement	Japan, ^(7)^
6	49/M	Lower lip cancer	Pembrolizumab	4 months	+	−	−	None	+	Improvement after relapse	Japan, Our case

Abbreviations: GC, glucocorticoid; HC, hydrocortisone; ICI, immune checkpoint inhibitor; M, male; MTX, methotrexate; NA, not available; PSL, prednisolone; TCZ, tocilizumab; +, present; −, absent.

We retrospectively analyzed the medical records of 713 patients with cancer who received anti-PD-1 therapy (pembrolizumab or nivolumab) at our hospital until March 2023 to evaluate the incidence of anti-PD-1 antibody-induced chondritis. This study was conducted with the approval of the ethics committee of Osaki Citizen Hospital (number: 20230605-7). RP occurred only in one (our case) of 303 pembrolizumab-treated patients during a median period of 31.1 (range: 1.1-73.5) months. Furthermore, tracheobronchial lesions are less frequent at the disease onset than auricular lesions, the most common features in RP ^(2)^. However, 5 of 6 (83.3%, Table 1) patients with ICI-induced RP initially manifested tracheobronchial chondritis without auricular involvement, indicating that ICI-induced RP is a more distinct clinical phenotype than RP unrelated to ICIs. Overall, these findings indicate that PD-1 inhibition-induced RP is unusual and atypical. In conclusion, physicians should recognize that this condition can occur as a rheumatic irAE in patients with cancer receiving anti-PD-1 therapy.

## Article Information

### Conflicts of Interest

None

### Acknowledgement

The authors would like to thank Editage (www.editage.jp) for English language editing.

### Author Contributions

TM, SC, TS, HS, and MK were involved in the clinical management of the patient, and KS evaluated the biopsy findings. TM drafted the manuscript. SC, TS, HS, KS, and MK critically reviewed the manuscript. All authors approved the final manuscript.

### Informed Consent

Written informed consent for publication was obtained from the patient.
